# Secure Cooperation of Autonomous Mobile Sensors Using an Underwater Acoustic Network

**DOI:** 10.3390/s120201967

**Published:** 2012-02-10

**Authors:** Andrea Caiti, Vincenzo Calabrò, Gianluca Dini, Angelica Lo Duca, Andrea Munafò

**Affiliations:** 1 Department of Energy and Systems Engineering, Inter-University Center on Integrated System for the Marine Environment, Interdepartmental Research Center ”E.Piaggio”, University of Pisa, Largo L. Lazzarino, 1, Pisa 56126, Italy; E-Mails: andrea.caiti@dsea.unipi.it (A.C.); v.calabro@dsea.unipi.it (V.C.); 2 Department of Information Engineering, Inter-university center on Integrated System for the Marine Environment, Interdepartmental Research Center ”E.Piaggio”, University of Pisa, Largo L. Lazzarino, 1, Pisa 56126, Italy; E-Mails: gianluca.dini@iet.unipi.it (G.D.); angelica.loduca@iet.unipi.it (A.L.D.)

**Keywords:** Autonomous Underwater Vehicles (AUVs), acoustic communication, network security, security and trust, adaptive systems, sensor networks, cooperative systems

## Abstract

Methodologies and algorithms are presented for the secure cooperation of a team of autonomous mobile underwater sensors, connected through an acoustic communication network, within surveillance and patrolling applications. In particular, the work proposes a cooperative algorithm in which the mobile underwater sensors (installed on Autonomous Underwater Vehicles—AUVs) respond to simple local rules based on the available information to perform the mission and maintain the communication link with the network (*behavioral approach*). The algorithm is intrinsically robust: with loss of communication among the vehicles the coverage performance (*i.e.*, the mission goal) is degraded but not lost. The ensuing form of graceful degradation provides also a reactive measure against Denial of Service. The cooperative algorithm relies on the fact that the available information from the other sensors, though not necessarily complete, is trustworthy. To ensure trustworthiness, a security suite has been designed, specifically oriented to the underwater scenario, and in particular with the goal of reducing the communication overhead introduced by security in terms of number and size of messages. The paper gives implementation details on the integration between the security suite and the cooperative algorithm and provides statistics on the performance of the system as collected during the UAN project sea trial held in Trondheim, Norway, in May 2011.

## 1. Introduction

Several recent researches have shown how a set of autonomous mobile agents and sensors, able to self-adapt and self-configure, can be used in several complex scenarios [1]. The collaborative use of multiple sensors is in fact of great advantage thanks to the resulting flexibility and robustness in the accomplishment of tasks. For instance, exploration of partially known or unknown environments can effectively be performed by a team of cooperating autonomous vehicles with an optimized use of the available resources and consequent saving of time and money [2]. As another example, the use of sensor networks for continuous monitoring of vital areas allows for disaster prevention and for a prompt reaction against unexpected situations [3].

One scenario in which the use of multiple vehicles or sensor nodes presents critical aspects and peculiar characteristics is the underwater one. Many relevant infrastructures are placed very close to the sea or directly in the water, opening new scenarios for the use of Autonomous Underwater Vehicles (AUVs) and the development of Autonomous Ocean Sampling Networks (AOSN), where multiple nodes can cooperate as a group to achieve some common goals [4].

Any cooperative mission necessarily involves communication among multiple agents. When the cooperating sensors are used in the underwater domain, communication issues become of paramount importance and in fact the agent spatial locations and mutual separation has a direct influence on the capability to communicate [5,6]. In the last years some studies have been carried out to include communication constraints into the development of cooperative strategies for set of vehicles [7,8]. However the impact of limited and/or unreliable communication has not been fully characterized. Most of the cooperation strategies proposed in the literature have been focused on cooperation of aerial or terrestrial vehicles, but these algorithms are not directly applicable in the underwater case due to the strong variation in space and time of the communication medium. Acoustic propagation, the main means of underwater communication, is strongly dependent on local environmental conditions, and during the evolution of the mission each vehicle can experience abrupt changes in the channel, with a consequent variation in communication performance. Moreover, acoustic communication is severely band-limited and range-limited. Sudden reduction of the channel capacity and bandwidth, or even a temporary loss of connectivity with the rest of the team, is a frequent condition for underwater communications.

In operative scenarios, not only is it necessary to share information to achieve the mission objectives, the ability to securely communicate also becomes a key issue so that the correct data is transmitted and received by the right agents, and only among the desired group. The possibility to share in a secure way the necessary information may in fact determine the success or the failure of the mission as a whole. Listening to private messages, or modification or injection of fake data are all usual threats in communication networks. They become even more critical in the context of distributed agents since cooperation may be achieved only when all the components receive the expected data from the legitimate peers. The underwater environment poses unique challenges also as far as network security is concerned, and again the traditional security mechanism successfully used and implemented on radio-based network suddenly becomes infeasible.

With this work we tackle the problem of secure cooperation of a team of mobile underwater sensors or AUVs within surveillance and patrolling applications. The contribution of the paper is two folds:

- A novel cooperative adaptive algorithm for mobile agents is proposed, with the goal of protecting an asset (e.g., a critical infrastructure such as a power plant placed on the shore or directly in the water) using detection sonars mounted on each agent. The algorithm takes explicitly into account communication constraints among the agents, in terms of maximum communication range achievable with a desired level of performance. It allows the vehicles to autonomously position themselves in order to cover the maximum area around the asset by means of the detection sonars, adapting their behaviour to the specific communication performance encountered as the mission proceeds. It is a distributed approach, since each vehicle takes local decisions in order to achieve the final goal, and it is based on the concept of *emergent behaviour* defining the action and the behaviour of each agent through simple elementary rules [9–11]. The algorithm allows for reconfigurability in response to oceanographic variations and/or external events. When a vehicle leaves or a new one joins, the vehicles may change their positions to adapt themselves to the new configuration so to achieve again the maximum asset protection guaranteeing the communication connectivity among the currently available agents.
- A set of network security solutions has been designed, functional to the cooperative strategy and tailored to the communication limitations of the medium. In contrast to traditional wired networks, an adversary equipped with an acoustic modem can easily eavesdrop as well as modify and insert fake messages [12,13]. In order to address these threats, we implement the cooperation algorithm according to the group communication paradigm. Vehicles in the group share a group key they use to encrypt and authenticate broadcast messages. Whenever a vehicle leaves the group, either because it has finished its mission or because it is (suspected to be) compromised, the current group-key is revoked and a new one distributed (forward security). Communication confidentiality and authentication is addressed with a cryptographic suite that keeps at minimum message expansion. This is done to cope with bandwidth and range limitation and to limit the amount of message overhead required by other cryptographic approaches. Furthermore, we use a secure rekeying protocol that is efficient from two standpoints: the number of rekeying messages is logarithmic in the number of vehicles; a rekeying message does not carry any additional proof of key authenticity so avoiding message expansion.

It is remarked that in this work the communication limitations in nodes cooperation are tackled from the application level point of view, *i.e.*, proposing a cooperation strategy that attempts to minimize the information exchange among the nodes. The strategy will be shown to be robust with respect to connectivity loss or communication range degradation. The aspects more related to the physics of underwater acoustic propagation have been discussed elsewhere [8], where it has been shown how to compute communication figures of merit (bandwidth, signal-to-noise ratio, communication range) from oceanographic environmental properties. In this paper, each node uses the maximum communication range as the available information on the communication channel properties. Finally, one more consideration can be done with respect to Denial of Service (DoS), one of the most severe threats against network availability in the underwater scenario. In fact, contrasting DoS in underwater networks is even more complicated than in traditional networks due to the intrinsic limitations of the acoustic channel [6]. The proposed security suite is aimed at communication integrity and confidentiality and thus does not provide any countermeasures against DoS. However, the cooperative algorithm is intrinsically reactive against DoS due to the emergent behaviour approach. Whenever an enemy succeeds in disrupting communication among vehicles, the simple rules that drive vehicle motion make them move closer to the asset in order to ensure protection regardless what the other agents are doing. As a result, a DoS attack effectively degrades the performance of the cooperation, but it cannot prevent to continue the mission with a limited number of cooperating nodes, or at the very least with all the nodes acting individually. In practical situations, if a DoS persists, as an extreme measure, an underwater node may surface and communicate with a land station using standard radio communication technologies. It is finally remarked that the tools proposed can be easily generalized to other underwater applications, though in the rest of the paper the reference applicative scenario is that of underwater surveillance.

In the paper, we report some results to characterize the application level performance of an underwater acoustic network including the cooperative strategy and the security suite proposed in the paper. Raw data were obtained during the experimental campaign UAN11 held in May 2011, within the Underwater Acoustic Network (UAN) project [14]. Field data show that the security solution is efficient in terms of number of messages and message size, reducing the time and the energy of the transmission.

The paper is organized as follows: in the next section the problem setting is described, defining in general terms the secure cooperative problem addressed. Section 3 describes the adaptive/cooperative algorithm for a team of autonomous underwater vehicles, abstracting from the specific network architecture chosen. Area coverage performance is described and the main algorithm limitations discussed. In Section 4 the secure communication procedures are presented. In Section 5 we report the result from the application of the algorithms during the UAN11 sea trial. Finally, conclusions are given in Section 6.

## 2. Model of the System and Problem Setting

**Figure 1.**
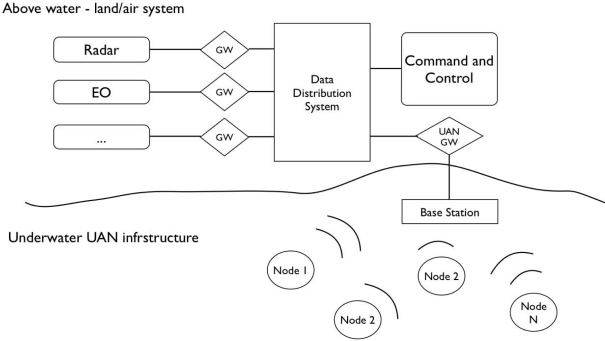
Conceptual overview of the UAN scenario: integration of above water and underwater systems.

The research presented is motivated by the framework of the European project UAN—Underwater Acoustic Network [14]. For this reason, without entering into details, it is important to define the general scenario to understand what is the background of the proposed methods and approaches. The main objective of the project is that of implementing a generic ad-hoc mobile acoustic network, composed by fixed and mobile nodes for underwater surveillance of off-shore and coastline critical infrastructures. The underwater network has to be integrated within a wider network, including above water nodes and sensors. Figure 1 gives a conceptual overview of the UAN scenario: the Land/Air system represents what can be thought as a traditional radio based sensing and communication infrastructure; the underwater part is composed by fixed and mobile nodes (AUVs) with detection and communication capabilities. The integration of the different systems has to be guaranteed by an appropriate communication and networking infrastructure in order to ensure the exchange of information among the various elements of the protection system. The UAN project hence has to deal with several aspects, including but not limited to:

- A deep analysis of the underwater communication physical layer in order to use existing or develop new acoustic technologies for efficient communication support and embedded signal processing;
- Development of tools for prediction of the acoustic channel performance, especially from a communication system standpoint;
- Development of reliable and efficient network architectures able to make the underwater communication as effective as possible. Traditional networking schemes are difficult to be taken to underwater scenarios: MAC schemes, routing protocols and network security features are all aspects that need to be investigated in order to find suitable solutions in situations characterized by long delays, frequency selectivity, and low bandwidth;
- Development of coordination strategies for the management of the mobile nodes of the network so that it is possible to dynamically place the nodes in order to maximize some communication related performance cost functions and periodically measure the environment; at the same time, the coordination strategy must guarantee the operation of the intrusion detection payload as appropriate.

The approaches presented in this paper are at the application level of the UAN project. While the experimental results have been obtained with the UAN-project implementation of the underlying layers [14], it is remarked that our algorithms can as well be applied with any alternative network infrastructure.

The envisaged scenario consists of:

- A land station which acts as a command and control (C2) center for the physical defense of a critical infrastructure;
- An underwater base station wired to the shore with a high bandwidth link. This station represents the connection between the above and below water environments; for this reason this element is both a part of the acoustic network and of a traditional wired communication infrastructure;
- Fixed and mobile nodes (n) acoustically connected in an underwater network which includes the base station. Each node is equipped with an on board sonar for intrusion detection and with an acoustic modem for communication purposes.

From the cooperative standpoint, the overall mission goal is that of covering with the vehicle detection sonars the largest possible area in the neighbourhood of a given asset where the land station also resides. Since the performance of the AUV devices depends on the local oceanographic conditions, we also assume that each agent or node is equipped with a sensor able to measure the environment variability (e.g., Conductivity, Temperature, and Depth—CTD probe) and with an acoustic channel performance predictor to convert these measurements into communication performance ([6,8]).

The cooperation among vehicles is modelled according to the group communication paradigm [15]. Vehicles may dynamically join and leave the group. A vehicle joins the group upon starting its mission. A node leaves the group, or is forced to, when the node has finished its mission, has been lost, or has been compromised (or believed so).

**Figure 2.**
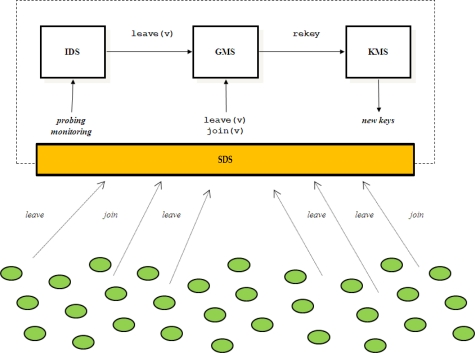
Group Controller.

**Figure 3.**
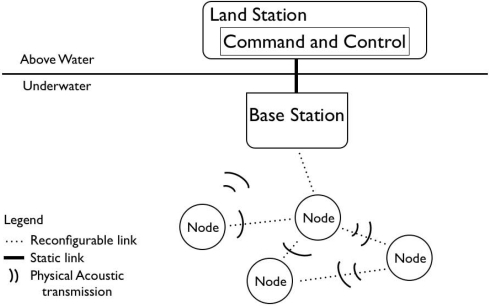
Architecture diagram with spatial configuration of various fixed and mobile nodes as well as the base station with the command and control center.

With reference to Figure 2, the group of vehicles is managed by a Group Controller (GC) that is composed of four main components: a Group Membership Service (GMS), a Key Management Service (KMS), an Intrusion Detection System (IDS) and a Secure Dispatching Service (SDS). The GMS maintains the membership of the group by keeping track of sensor nodes that join and leave the group. The IDS probes/monitors network activities and vehicles behaviour to detect compromised vehicles [16]. Upon detecting a compromised sensor node, IDS forces the vehicle to leave the group by invoking the GMS leave operation and specifying the vehicle identifier as argument. Whenever a sensor node leaves, or is forced to leave the group, the group-key is renewed in order to guarantee the backward and forward security requirements. KMS is the component that is responsible to perform rekeying. Upon handling a change in the group membership, GMS invokes the KMS rekeying operation. Furthermore, KMS also performs periodic rekeying aimed at reducing the amount of material encrypted with the same key available to an adversary. Finally, the SDS provides a cryptographic suite for message encryption and authentication. In UAN, the GC is implemented by the Base Station (see Figure 3) that has plentiful of resources and will not be compromised. In the rest of the paper we focus on the KMS and SDS components.

## 3. Cooperative Algorithms for Mobile Sensors

This section focuses on the development of an adaptive cooperative algorithm for mobile sensor nodes, the moving components of the underwater network. When fixed sensors are present they will participate to the global performance, in terms of detection and communication, but since their position is fixed they cannot reconfigure and adapt themselves to varying scenarios. In this case, it will be the mobile agents’ responsibility to consider the presence of the fixed nodes, so as to guarantee that the final goal can be reached. Moreover, in order to allow a simpler description of the proposed method, in the remainder of this section, we assume the existence of an appropriate communication infrastructure which is reliable and robust enough to effectively allow a team of mobile agents to securely communicate and collaborate to achieve the required common goal. Further details on these aspects are given in Section 4.

The main mission objective is that of covering with the mobile node detection sonars the maximum area around the asset, while each vehicle has to move to keep at least one other vehicle of the team within its communication range. This general goal is hence divided, according to the *behavioral approach* paradigm, into simpler subtasks (behaviors or rules) solvable in parallel [11]. A composition rule is also defined to transpose the commands generated by each subtask into one single motion command for each vehicle.

Let us assume we have the availability of *n* AUVs, each one equipped with an acoustic modem for communication up to a maximum range *R_C_* and with a detection sonar characterized by a maximum range *R_D_*. We consider the *i − th* vehicle as defined by the discrete kinematic model:

(1) $$x^{i}(k+1)=x^{i}(k)+T_{s}u^{i}(k)$$

where *x^i^*(*k*) ∈ ℜ^3^ is the i-th vehicle location at time *k*, *u^i^*(*k*) ∈ ℜ^3^ is the control input to be defined and *T_s_* is the sampling period. The above-mentioned objective is hence split into two subtasks or rules:

1. Move toward the High Value Asset (HVA) to be defended.
2. Move away from your closest neighbor but without exiting from its communication range.

The first task allows the vehicle to move closer to the asset to ensure the asset protection. The second task represents the coordination level. It allows each agent to adapt its movement to keep into account actions of the other members of the team. Specifically, it lets each vehicle cover the maximum area around the asset, with minimum overlaps of the on board sonar detection ranges, while guaranteeing the communication links with at least one other teammate. The composition of the two subtasks is achieved through a priority-based mechanism which assigns to each of the subtask a dynamic priority on the basis of the vehicle status with respect to the fulfillment of each one of the subtasks.

**Figure 4.**
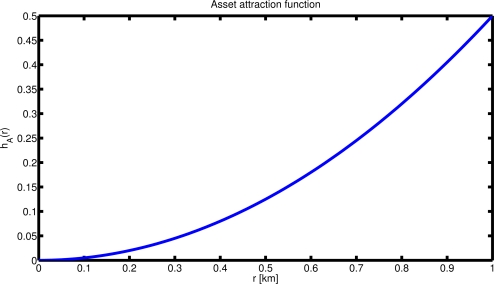
Function of interest for task 1: move towards the asset to be protected. The higher the distance from the asset the higher the interest in fulfilling the task.

**Figure 5.**
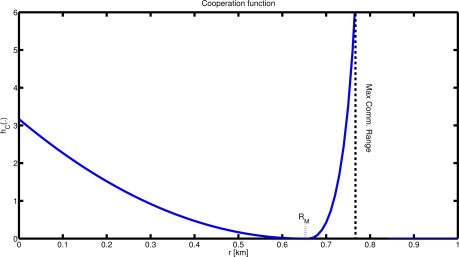
Interest function for task 2: move away from your closest neighbour while maintaining the communication connectivity. The function represents the cohesiveness among the vehicles, as a function of range from the nearest neighbour (move away when the vehicles are close, move closer for values approaching the maximum communication range).

In particular, each vehicle assigns to each task a priority computed on the basis of an *interest function*. This function defines, at any given stage of the mission, the interest of the vehicle in fulfilling the specific task while a comparison among the functions of interest determines the priority of the tasks to be executed at any time frame by each vehicle:

- The *Asset attraction function*, *h_A_*(*x_asset_*, *x^i^*(*k*)) is a function of the agent’s distance from the asset *x_asset_*. It defines the interest of the agent (priority of the subtask) in moving towards the asset (see Figure 4). Its parametric definition is as follow:
  (2) $$h_{A}\left(x_{\mathrm{asset}},x^{i}(k)\right)=\frac{{\left\|x_{\mathrm{asset}}-x^{i}(k)\right\|}_{2}}{N}$$
  where *N* is a positive constant.
- The *Coordination function*, *h_C_*(*x^j^*(*k*), *x^i^*(*k*)) defines the priority of the coordination task (see Figure 5). It is computed online and modified by the vehicle during the evolution of the mission on the basis of the detection and communication performance of its onboard devices: the detection sonar range *R_D_* defines the minimum distance between two vehicles; the maximum communication range *R_C_* achieved at a given spatial and temporal location defines the maximum separation between two vehicles; in addition, we also define the parameter *R_M_* as the maximum distance at which each agent wants to keep its closest neighbor. The parameter *R_D_* can be thought as the maximum detection range at which the detection performance is above a desired threshold $\bar{\mathrm{TH}}$, *R_M_* as the range above which the detection performance is below a minimum level *TH*. The coordination function is defined as:
  (3) $$h_{C}(x^{j}(k),x^{i}(k))=\{\begin{array}{ll} \frac{q\left(R_{M}-{\left\|x^{j}(k)-x^{i}(k)\right\|}^{2}\right)}{2} & \text{if}\;\left\|x^{j}(k)-x^{i}(k)\right\|\leq R_{M}\\ \frac{Q}{R_{M}-\left\|x^{j}(k)-x^{i}(k)\right\|}-\frac{\left\|x^{j}(k)-x^{i}(k)\right\|}{{\left(R_{C}-R_{M}\right)}^{2}}+C & \text{if}\;R_{M}\leq \left\|x^{j}(k)-x^{i}(k)\right\|\leq R_{C}\\ 0 & \text{othersie} \end{array}$$
  where *q* and *Q* are positive constants and $C=-\frac{{2R}_{M}-R_{C}}{{\left(R_{M}-R_{C}\right)}^{2}}$ is a smoothing constant.

The steps of the cooperative algorithm are now summarized. The algorithm, described in what follows for the i-th one, is the same for every vehicle:

1. Vehicle *i* receives from all the connected nodes their locations and their maximum detection sonar range;
2. Vehicle *i* selects its closest neighbor;
3. Assign a priority to each task as defined by the corresponding interest function (Equations (2) and (3));
4. Compute the overall velocity control applying (Equation (4)) and move accordingly.

Finally, the agent control input *u*(*t*), at each time frame is computed as the vector sum of the gradient of each interest function:

(4) $$u(t)=u_{A}(t)+u_{C}(t)=▽h_{A}+▽h_{C}$$

Note that the algorithm makes each vehicle able to move back to the asset it needs to protect even when it loses the communication with the other team members, since each agent can always execute task 1 (move towards the asset). In this way the algorithm becomes intrinsically robust against a DoS attack and even when a vehicle cannot communicate with the rest of the team it can always move to ensure the protection of the asset (e.g., vehicle equipped with appropriate deterrence means). The performance of the cooperation is degraded but the subset of the agents that can still communicate, or in the worst case, each vehicle independently, can continue the mission. In addition, at each step of the mission each agent does not necessarily need to receive information from all other nodes: only information from its closest neighbour is needed for the coordination task. If information is available from more than one vehicle, an additional step is needed to determine the closest neighbour. In any case, the amount of information the vehicles need to exchange is limited, as they only require communicating their own position and maximum detection sonar range. Finally, it is worth pointing out that when a vehicle loses the communication with the remainder of the team, it may still be able to execute task 2 but using only not up-to-date information. In this case, the agent calculates the control input (Equation (4)) on the basis of the last known position of its neighbours.

### 3.1. Area Coverage Performance and Algorithm Limitations

In this section the area-coverage performance of the algorithm are shown with respect to optimal geometrical solutions. The total sonar coverage depends on the sonar detection range *R_D_* and on the maximum distance allowed *R_M_* between two vehicles.

**Figure 6.**
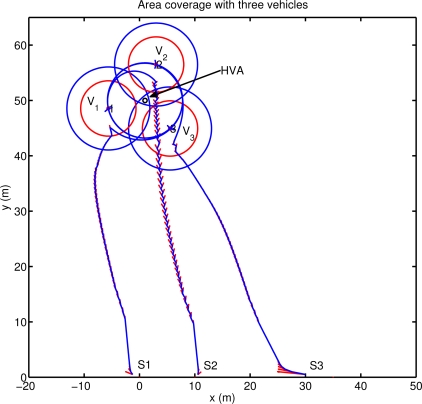
Area coverage performance with three vehicles. The optimal locations for the agents to obtain the total coverage of a circle of radius *R_M_* are the vertices of the inscribed triangle. A circular motion is stimulated when each vehicle communicates its foreseen location at its next communication step. For each agent, bold lines are used to depict both the maximum distance allowed *R_M_* and the detection sonar maximum range *R_D_* (*RM* > *RD*). Red arrows indicate the vehicles speed.

**Figure 7.**
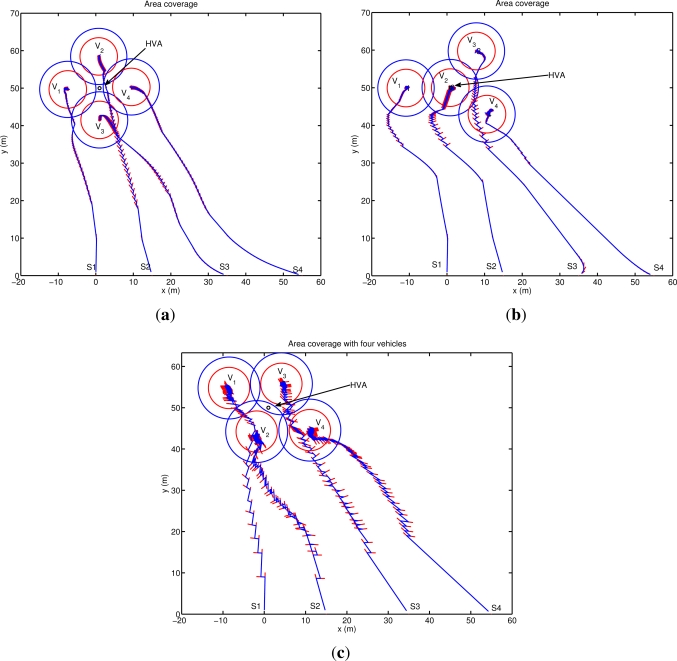
Area coverage with four vehicles. Increasing the number of vehicles different configurations can be obtained depending on the relative weight given to the two tasks. Top-Left: symmetric configuration obtained for *N* = 20 in *h_A_*. Top-right: configuration with one of the vehicle over the high value asset (*N* = 10). Bottom: asymmetric configuration obtained by increasing the priority of task 2. As in the three vehicles case, the stability of this final stable configuration is related to the information communicated among the team members. In this case, when each vehicle communicates its foreseen location at its next communication step the asymmetric configuration becomes unstable and the group alternates between a configuration where *V*_1_ and *V*_4_ are further away than *V*_2_ and *V*_3_ and another one in which *V*_1_ and *V*_4_ are closer than *V*_2_ and *V*_3_ to the asset. For each agent, bold lines are used to depict both the maximum distance allowed *R_M_* and the detection sonar maximum range *R_D_* (*RM* > *RD*). Red arrows indicate the vehicles speed.

**Figure 8.**
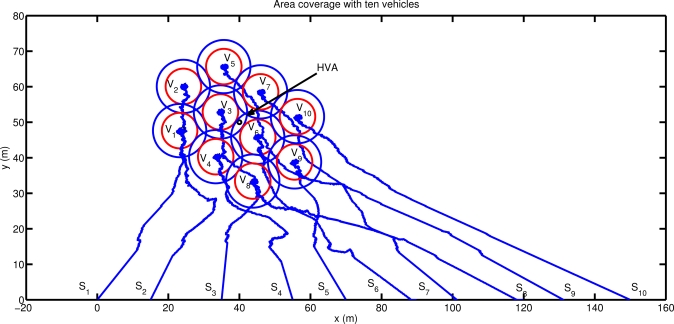
Area coverage performance with ten vehicles. The HVA is placed at the center of the sonars detection area.

Figure 6 shows the result obtained applying the proposed method using three vehicles. The agents place themselves in order to position the asset at the center of the detection area. The final configuration reached allows the vehicles to completely protect a circle of radius *R_M_* around the asset with a minimum overlap (*R_M_* − *R_D_*) of the vehicle sonars, and with a detection level always greater than the minimum desired. The final agent positions coincide with the analytical solution of the area coverage problem of a circle of radius *R_M_*, with the vehicles placed on the circumference, as vertices of the equilateral triangle inscribed in the circle itself. The configuration reached has three axes of symmetry, one per each vehicle, and along these axes the detection coverage is more effective (detection directivity). It is interesting to note that the stability of the final configuration reached is related to the information communicated among the team members. In particular, if each vehicle communicates to the remainder of the team its foreseen location at its next communication step after applying the proposed motion coordination algorithm, then a circular motion around the asset is automatically stimulated and the vehicles periodically scan the whole area around the asset. Note in fact that, for symmetry reasons, all the configurations with the asset placed at the center of mass of the team detection area are all optimal. Increasing the number of vehicles, the area that can be protected becomes larger and different solutions can be obtained depending on the relative weight given to the two tasks. Simulations are performed for a four agents case and for a more complex scenario with ten vehicles. Figure 7 shows three configurations reached using four agents. In the first case (Figure 7(a)) the configuration is symmetric with the asset placed at the center of mass of the vehicles’ detection area. In this case (obtained setting *N* = 20 in *h_A_*, *q* = 1 in *h_C_*) the agents are placed as vertices of a square around the asset, which is however located inside an area characterized by a lower level of detection (*i.e.*, asset further than *R_M_*). Increasing the interest of the vehicles in moving towards the asset (*i.e.*, increasing the priority of task 1 with respect to task 2: *N* = 10 in *h_A_*) the configuration of Figure 7(b) can be reached: one of the vehicles is located directly on the high value asset to protect while the remainder of the agents reach a final stable configuration around it. In this case, the locations of the external agents in the final configuration depend on their initial conditions (all the points around the vehicle in the middle are equally attractive). Increasing the priority of task 2 (*q* = 100 in *h_C_*) yet another configuration can be obtained. The vehicles are taken to an asymmetric final configuration characterized by detection directivity. As shown in Figure 7(c), the asset is always placed at the center of the sonar detection area, and the vehicle positions coincide with the vertices of a regular polygon around the asset to be defended. In Figure 7(c), agents *V*_1_ and *V*_4_ are further away from the asset than vehicles *V*_2_ and *V*_3_ providing the increase in directivity. Again, as in the three vehicles case, the stability of the final configuration is related to the information communicated among the team members. In this case, when each vehicle communicates its foreseen location at its next communication step, the asymmetric configuration becomes unstable and the group alternates between a configuration where *V*_1_ and *V*_4_ are further away than *V*_2_ and *V*_3_ and another one in which *V*_1_ and *V*_4_ are closer than *V*_2_ and *V*_3_ to the asset. Finally, as a more complex scenario, ten vehicles are simulated. Figure 8 shows the agent path and the final formation obtained, with the HVA placed at the center of the vehicles detection area. The agent locations around the asset depend on their initial positions and, as the number of vehicles increases, it becomes more complicated to characterize the final distribution of agents. A theoretical analysis of such a problem is however beyond the scope of the paper.

As shown through simulations the proposed method is able to solve the general area coverage problem, reducing the overlap of sonar detection sonars and ensuring that each agent maintains at least one other vehicle in its communication range. The approach, which is suited to be implemented on underwater vehicles, requires little communication among the vehicles. Furthermore, with limited modifications on the type of information transmitted but without introducing additional data exchange or algorithmic complexity, it instigates additional and more complex team behaviors (e.g., patrolling).

The resulting behavior of the team of AUVs is strongly dependent on the parameters of the interest functions associated with each rule of the algorithm. The slope of the functions, in fact, determines the strength of each rule and their relative weight, at each time step. A steep asset attraction function (*h_A_*) implies an increase in the attractive field of the HVA while a modification in the shape of the cooperation function relates to the detection sonar overlap. For instance, a more gentle slope for *h_C_*(.) produces, on one hand, an increase in the sonars overlap, reducing the total area coverage, but, on the other hand, it increases the total detection level, as it can be computed using, for example, the approach proposed in [17]. The selection of the specific parameters is hence a design tradeoff and depends on the specific application. The flexibility of the proposed algorithm, based on the selection of appropriate interest functions to obtain desired global behaviors, represents, at the same time, its main limitation. It may in fact be difficult to tune the function parameters to achieve the desired mission goal and in the cases treated here the choice was based on empirical rules. Further investigations are ongoing to analytically guarantee the optimality of the selection. While different approaches exist in literature on the area coverage problem by means of a network of cooperating sensors/agents (see for example [18] and [19]), they are, however, designed for reliable radio-based networks and for isotropic propagation media, often assuming unlimited communication ranges. Even in the cases where communication constraints are explicitly considered [20], they are typically assumed as indirected and homogeneous. On the contrary, the approach proposed in this work has been devised from the beginning to be able to tackle communication and detection variations, and specifically tailored to the characteristics of the underwater channel. The cooperation interest function *h_C_* is, in fact, computed on the basis of the detection and communication performance encountered as the mission proceeds, and it may be made dependent on the direction, allowing for the inclusion of detection and communication directivity.

## 4. Secure Cooperation

The cooperative algorithms of the previous section critically relay on the trustworthiness of the transmitted information. This is achieved by means of two services, the Secure Dispatching Service (SDS) and the Key Management Service (KMS). The SDS is responsible for protecting confidentiality and authenticity of messages by encrypting and decrypting them as well as generating and verifying proofs of their authenticity. The KMS is responsible for revoking the current key and distributing a new one either periodically or upon a vehicle leaving.

Implementing these services in a underwater acoustic network is challenging due to the severe limitations of the networking environment in terms of very high message propagation delay, very low bandwidth, and high energy consumption for communication. Limitation in the message size is hence of paramount importance in order to reduce battery consumption in autonomous nodes.

### 4.1. Secure Dispatching Service

The SDS implements the cryptographic transformations that have to be applied to network traffic segments. A cryptographic transformation specifies the cryptographic processing to be applied to messages before sending or after receiving them. For instance, in order to guarantee both confidentiality and integrity of a message, a possible transformation is *E_e_*(*m*||*h*(*m*)), where *E* specifies a symmetric cipher, *h* specifies an hash function and *e* is an encryption key. Alternatively, a transformation aimed at guaranteeing sole authentication of message *m* is *m*||*H_a_*(*m*), where *a* is an authentication key and *H* is a Message Authentication Code (MAC). The cryptographic suite, namely the actual cryptographic primitives that are used in a transformation, must be properly chosen because of the communication overhead it may imply.

#### 4.1.1. Confidentiality

Block ciphers split cleartext in blocks of fixed, predefined bit-length. In the most general case, cleartext length is not multiple of the block length. Thus padding is necessary. However, padding has the negative effect that the ciphertext may result up to one block longer than the corresponding cleartext. This effect is called ciphertext expansion. While ciphertext expansion is negligible in a traditional network, it becomes relevant in wireless sensor networks and, in particular, underwater acoustic networks. In these networks, communication and energy limitations require keeping a message size small and ciphertext expansion may introduce an overhead that is not negligible anymore. Let us consider the cooperation algorithm. At each step a vehicle sends 104-bit payload specifying the vehicle identifier (8 bits), position (two real numbers for a total of 64 bits), and sonar detection range RD (a 32-bits real number). Encrypting the payload by means of AES, whose block size is 128 bits, would introduce 24 bits of padding thus causing a 23% of payload expansion. Of course, for any given payload, the percentage weight of the ciphertext expansion much depends on the block size and ultimately on the cipher. However, in order to completely avoid the ciphertext expansion problem, we have used the CipherText Stealing (CTS) technique that alters the processing of the last two blocks of plaintext, resulting in a reordered transmission of the last two blocks of ciphertext and no ciphertext expansion [21].

#### 4.1.2. Authenticity in UAN

Encryption without authentication is insecure [22]. For example, an adversary may flip bits in unauthenticated ciphertext and cause predictable changes in the plaintext that receivers are not able to detect. To address this vulnerability, UAN always authenticates messages, but encryption is optional. Message confidentiality is necessary only when there is some information that has to be kept secret. UAN uses MACs to address authentication only. In contrast, it uses the transformation *E_e_*(*m*||*h*(*m*)) when it addresses both encryption and authentication [22]. More specifically, UAN uses SHA-256 both as hash function and to build keyed hash functions (HMAC) [22,23]. Security of hash functions is directly related to the length of the digest. However, as a digest is appended to the message, it becomes another source of message expansion and consequent communication overhead. For instance, SHA-256 has a 256-bit output, which is about 2.46 times the size of the payload of cooperation algorithm messages. For this reason, UAN features a trade-off between security and performance by using 4 bytes digests resulting from truncating the real hash function value. Using such a short hash function value is not detrimental to security [24]. An adversary has 1 in 2^32^ chances to blindly forge a digest. If an adversary repeatedly tries to forge it, he/she needs 2^31^ trials on average. However, the adversary cannot perform trials off-line. This means that the adversary has to validate a given forgery only by sending it to an authorized receiver. This implies that the adversary has to send 2^31^ messages in order to successfully forge a single malicious message. In a conventional network this number of trials is not large enough. However, in a underwater acoustic network this may provide an adequate level of security. An adversary can try to flood the network with forgeries, but on a 2 kbps channel with 184-bit messages, he/she can only send about 11 attempts for second. Thus, sending 2^31^ messages requires around 75 months, *i.e.*, about 6 years. Battery-operated vehicles have not enough energy to receive that many messages. Furthermore, the integrity attack would translate into a denial of service attack since the adversary needs to occupy the acoustic channel for a long time. Fortunately, it is feasible to detect when such an attack is underway. UAN uses a simple heuristic: vehicles could signal the base station when the rate of digest/MAC failures exceeds some predetermined threshold.

### 4.2. Key Management Service

**Figure 9.**
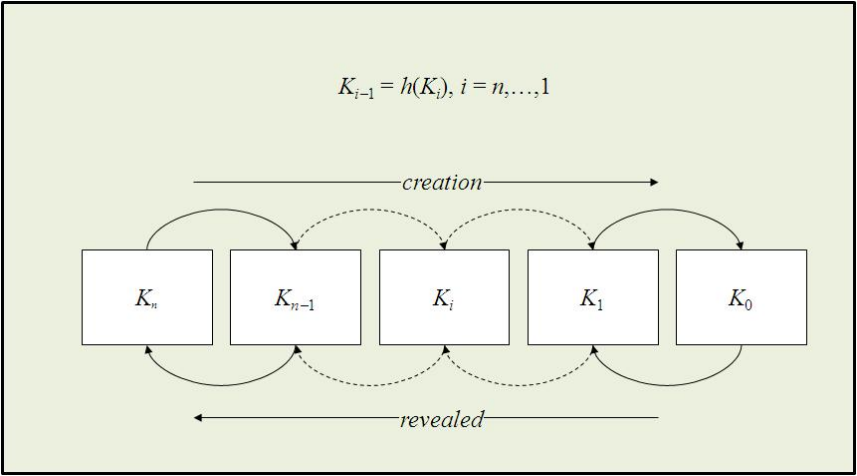
The hash chain.

**Figure 10.**
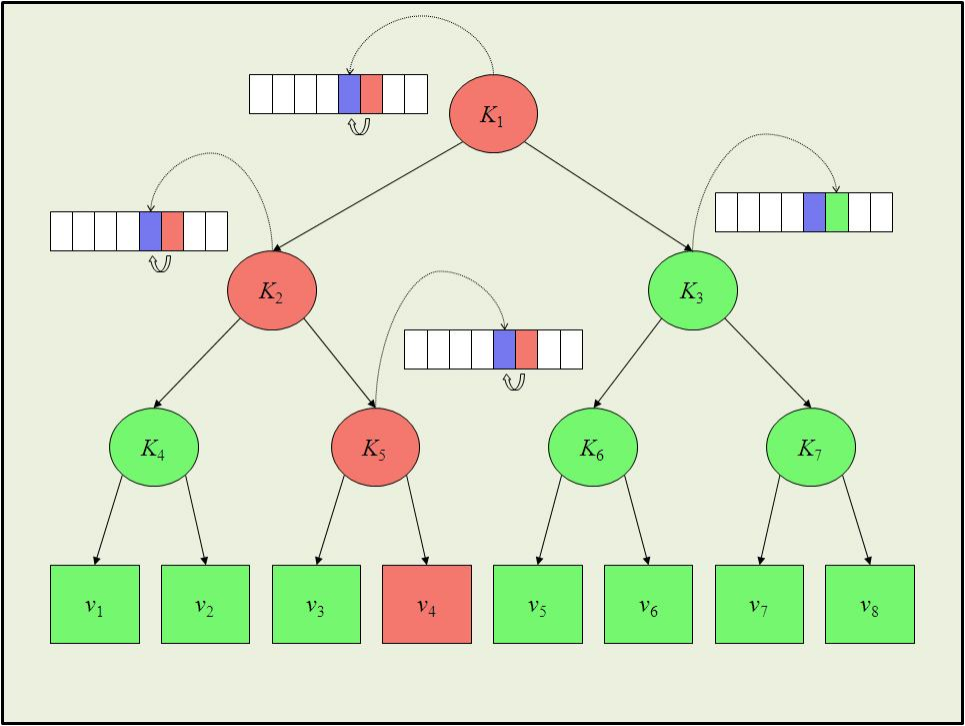
The key tree.

Each time a node leaves the system, the KMS generates and distributes a new group key. This is done to avoid that an old vehicle is able to read new messages. The scalability of the rekeying service depends on the chosen rekeying protocol. In UAN, we chose S2RP, a secure and scalable rekeying protocol for resource-constrained devices [25]. S2RP is particularly suitable for UAN for two reasons. First of all, S2RP provides a very efficient proof of key authenticity. Actually, S2RP verifies the authenticity of a key by computing a hash function. So, verification is very computing efficient and does not require any additional information, e.g., MACs or digital signatures, which would cause message expansion. Secondly, S2RP requires a number of rekeying messages that is logarithmic in the number of vehicles, thus making the key distribution phase highly scalable. In short, the key authentication mechanism levers on key-chain, a technique based on the Lamport’s one-time passwords. A key-chain is a set of symmetric keys so that each key is the hash pre-image of the previous one (see Figure 9). Hence, given a key *K*^(*i*)^ in the key-chain, anybody can compute all the previous keys *K*^(*j*)^, *j* ≤ *i*, however nobody, but the key-chain creator, can compute any of the next keys *K*^(*j*)^, *j* > *i*. Keys are revealed in the reversed order with respect to creation order. Given an authenticated key in the key-chain, anybody can authenticate the next revealed keys by simply applying an hash function. For example, if *K*^(*i*)^ is an authenticated key, than anyone can verify the authenticity of *K*^(*i*+1)^ by verifying that *K*^(*i*)^ = *h*(*K*^(*i*+1)^). To reduce the communication overhead, KMS maintains a logical key tree (see Figure 10). Each internal node contains a key-chain, whereas each leaf is associated with a vehicle and contains the vehicle-key, *i.e.*, the secret key that the vehicle shares with KMS. We call current key of a key-chain the last revealed key of the key-chain and next key the hash pre-image of that key. Furthermore, we denote by *K_i_* and $K_{i}^{+}$ respectively the current and next key of the key-chain associated to tree node i. Notice that $K_{i}=h\left(K_{i}^{+}\right)$. Each vehicle maintains a *key-ring* that contains every key *K^i^* such that the sub-tree rooted at node i contains the leaf associated with the vehicle-key. Hence, with reference to Figure 10, the key-ring of vehicle *v*_4_ is {*K*_1_, *K*_2_, *K*_3_}. As it turns out, key *K*_1_, associated to the key tree root, is shared by all vehicles and acts as the group-key.

Let us now assume that vehicle *v*_4_ leaves the group. All keys in its key ring are considered compromised and KMS has to broadcast the respective next keys $\left\{K_{1}^{+},K_{2}^{+},K_{3}^{+}\right\}$ by means of the following rekeying messages:

1. *KMS* → *v*_3_ : $E_{K_{3}}\left(K_{5}^{+}\right)$
2. *KMS* → *v*_3_ : $E_{K_{5}^{+}}\left(K_{2}^{+}\right)$
3. *KMS* → *v*_1_, *v*_2_ : $E_{K_{4}}\left(K_{2}^{+}\right)$
4. *KMS* → *v*_1_, *v*_2_, *v*_3_ : $E_{K_{2}^{+}}\left(K_{1}^{+}\right)$
5. *KMS* → *v*_5_, *v*_6_, *v*_7_, *v*_8_ : $E_{K_{3}}\left(K_{1}^{+}\right)$

Upon receiving a rekeying message, after it has been properly decrypted, the authenticity of the next key therein contained is verified by computing its hash and comparing the result to the corresponding current key. For instance, upon receiving rekeying message 5, *v*_6_ decrypts the message by means of *K*_3_ and verifies the authenticity of $K_{1}^{+}$ by ascertaining that $K_{1}=h\left(K_{1}^{+}\right)$. As it turns out the rekeying protocol requires *O*(*logn*) rekeying messages, where *n* is the number of vehicles. Furthermore, given the key-chain authentication mechanism, every rekeying message needs to carry only the next key (in its encrypted format). No additional information proving key authenticity is thus required. Notice that this is a great advantage in terms of communication overhead with respect to using digital signatures, for example. Let us assume that group keys are 128-bit long and we use ECC-180 digital signature to authenticate them. ECC-180 is nowadays considered as secure as RSA-1024. In ECC-180 a digital signature is 360 bits and thus a rekeying message would be 488 bits, *i.e.*, 3.8125 times longer than in the approach proposed here.

## 5. UAN11 Field Test

**Figure 11.**
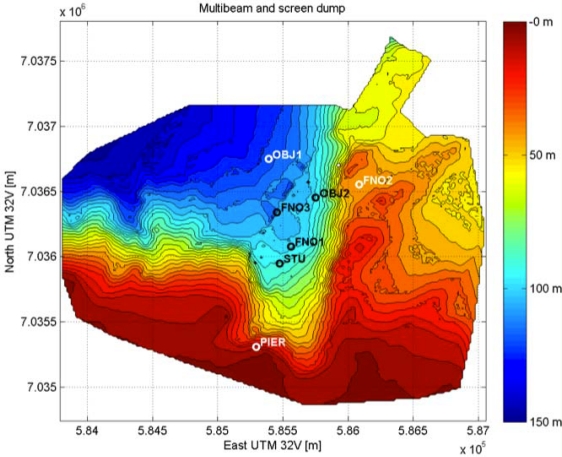
Test area during UAN11 experiment activity. The network topology is superimposed on the bathymetric lines of the area. The STU node represents the UAN base-station; FNO1, FNO2, FNO3 are the three fixed nodes; OBJ1 and OBJ2 indicate the locations of a simulated intruder. PIER represents the UAN land station with the command and control.

**Figure 12.**
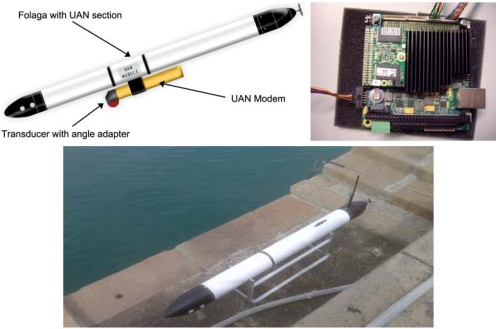
**Top left**: implementation of the proposed methodologies within the UAN project. The eFolaga vehicle [29] will carry a specific UAN payload section, with the UAN acoustic modem (developed by UAN partner Kongsberg Maritime) and electronic hardware in the dry section. **Top right**: the internal hardware on which the UAN system architecture has been implemented: a SECO104-CX700M board, equipped with a 1 GHz processor, 1 GB of RAM and 4 GB flash disk. **Bottom**: the eFolaga on the pier before an engineering test. The UAN section is inserted at the junction visible at mid-vehicle.

This section report results from the application of the above mentioned algorithms to the UAN project experimental activities held in Trondheim, Norway, in May 2011. The network (see Figure 11) was composed by up to four fixed nodes including the base station, two AUVs of Folaga class and one additional mobile node set-up on the Research Vessel Gunnerus using a transducer located at about 20 m depth. The command and control was located on shore at about 800 m from the base station. To give an idea of the tested network characteristics a brief description of the UAN architecture is now provided. Each node was equipped with an acoustic modem developed by UAN partner Kongsberg and capable of transmitting up to a rate of 500 bps. The network architecture used a CSMA protocol and the FLOOD routing algorithm [26] both directly implemented in the modems, an IP tunneling mechanism to establish the IP connection and UDP as transport protocol. Finally, the communication among the vehicles was achieved through the publish/subscribe system MOOS [27]. The most recent development of the Folaga class vehicle [28,29], was used during the experiment with a dedicated payload section which included the Kongsberg acoustic modem (electronics and transducer), and the system architecture previously described was implemented over a PC104 SECO104-CX700M board, based on a 1 GHz processor, 1 GB of RAM, a 4 GB flash disk (see Figure 12), running Linux Ubuntu 9.10. This board represents the high-level control element of each vehicle, and it is hardwired (RS232) to the acoustic modem for the communication with the other agents. Finally, the board controls the behaviour of the vehicle by communicating, through an Ethernet link, with a low-level controller which acts on the vehicle motors.

**Figure 13.**
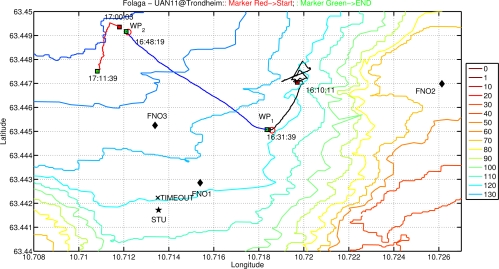
Folaga path during the experimental activity on May 27. In the first part of the day the vehicle was acoustically controlled by the UAN command and control center to proceed to deeper investigation in an area where an intruder was detected. However, the C2 moved the vehicle too far from the network where it lost the connectivity. According to the behavior described in Section 3 the vehicle autonomously planned a new mission to move towards the high value asset (the base station) to protect where it could re-enter the network.

**Figure 14.**
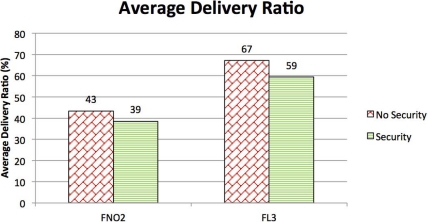
ADR performance.

The presence of such a mobile agent allowed for change in the network geometry to tackle changes in the environment. During the test, which involved several different communication objectives not covered in this work as they would go beyond the scope of the paper, the vehicles were used as data-relays to improve and to re-establish broken communication links between fixed nodes (network adaptivity to changing environmental conditions) and they were integrated into the UAN wide-area protection system for the protection of a high value asset co-located with the UAN base-station. Figure 13 shows a zoomed view of the network and the Folaga path in the afternoon of May 27. In the first part of the day the vehicle was acoustically controlled by the UAN command and control center with the objective of patrolling inside an area where an intrusion was suspected. However, the C2 set up a waypoint for the vehicle (WP2) which was too far away from the remainder nodes of the network and the vehicle lost the connectivity. According to the behavior described in Section 3 the vehicle autonomously planned a new mission to move towards the high value asset (the base station) to be protected where it could in fact re-enter the network. At this point the command and control took over the control of the vehicle again sending a new mission (manually aborted on the spot to proceed with other communication tests and hence not shown in the picture). During the experiment, the network was tested at first without the security features activated and then with the cryptography, integrity and authentication services enabled. Figure 14 shows a comparison in terms of Average Delivery Ratio (ADR). The ADR is defined as the average ratio between the number of received messages by a node and the number of sent messages to that node. It is clear from the picture that when the security was activated there was a decrease of 8% in the ADR. This decrease is due to two concurrent effects:

- The message expansion caused by the authenticator which in turn increases the probability of packet loss.
- A decrease in the acoustic communication conditions.

Even though it is not possible to evaluate the specific weight of each of the two components in the mix, the ADR decrease is sustainable and the effect of the use of network security appears not to be critical with respect to the decrease in performance due to the degradation of the communication channel.

## 6. Conclusions

The paper has described a methodology for secure cooperation within a network of autonomous mobile underwater sensors connected through an acoustic communication network. A cooperative algorithm based on the behavioural paradigm has been illustrated. Each mobile sensor solves simple parallel subtasks responding to local rules based on the available information to perform the mission and maintain the communication links within the network. The algorithm has been designed to be intrinsically robust, in the sense that with loss of communication among the vehicles the coverage performance (*i.e.*, the mission goal) is degraded but not lost. Moreover, the algorithm attempts to minimize the information exchange among the vehicles prior to the local decision. Area coverage performance of the proposed adaptive approach has been discussed and compared with those obtainable with optimal geometrical solutions. It was also shown how, in dependence with the type of information transmitted but without introducing additional data exchange or algorithmic complexity, the proposed algorithm may instigate additional and more complex team behaviors. Crucial for the agents cooperation is the trustworthiness of the messages among the sensors. To ensure it, a security suite based on the group communication paradigm has been designed, at the communication middleware level. The security suite has been specifically oriented to the underwater scenario, and in particular to the goal of minimizing message size overload and computational and message exchange increase, while still guaranteeing security. Details on the implementation of the given methodologies have been given, describing the architecture developed to integrate the cooperative algorithm into the security suite. Furthermore, we reported statistics and figures of merit on the performance of the UAN project underwater acoustic network as tested in May 2011, in the Trondheim area, Norway. The UAN experimental testing was focused on the general validation of the network architecture and it included all the security mechanisms described in this work and the autonomous adaptation and cooperation of the nodes.
